# ECMO-Unterstützung während der ersten 2 Wellen der Coronapandemie – eine Umfrage an Zentren mit hohen Fallzahlen in Deutschland

**DOI:** 10.1007/s00063-022-00951-3

**Published:** 2022-09-08

**Authors:** Alexander Supady, Guido Michels, Philipp M. Lepper, Markus Ferrari, Jens Wippermann, Anton Sabashnikov, Holger Thiele, Marcus Hennersdorf, Tobias Lahmer, Udo Boeken, Jan Gummert, Eike Tigges, Ralf M. Muellenbach, Tobias Spangenberg, Tobias Wengenmayer, Dawid L. Staudacher

**Affiliations:** 1 Interdisziplinäre Medizinische Intensivtherapie (IMIT), Universitätsklinikum Freiburg, Medizinische Fakultät, Albert-Ludwigs-Universität Freiburg, Hugstetter Str. 55, 79106 Freiburg, Deutschland; 2 Abteilung für Kardiologie und Angiologie I, Universitäts Herzzentrum Freiburg Bad Krozingen, Universität Freiburg, Freiburg, Deutschland; 3 Heidelberg Institute of Global Health, Universität-Heidelberg, Heidelberg, Deutschland; 4 Klinik für Akut- und Notfallmedizin, St.-Antonius-Hospital Eschweiler, Eschweiler, Deutschland; 5 Klinik für Innere Medizin V – Pneumologie, Allergologie und Intensivmedizin, Universitätsklinik des Saarlandes, Homburg/Saar, Deutschland; 6 Klinik für Innere Medizin I, Helios Dr. Horst Schmidt Kliniken Wiesbaden, Wiesbaden, Deutschland; 7 Universitätsklinik für Herz- und Thoraxchirurgie, Medizinische Fakultät, Otto-von-Guericke Universität Magdeburg, Magdeburg, Deutschland; 8 Herzzentrum, Klinik und Poliklinik für Herzchirurgie, herzchirurgische Intensivmedizin und Thoraxchirurgie, Universitätsklinikum Köln, Köln, Deutschland; 9 Herzzentrum Leipzig, Universitätsklinik für Kardiologie, Leipzig, Deutschland; 10 Medizinische Klinik I, SLK-Kliniken Heilbronn GmbH, Heilbronn, Deutschland; 11 Klinik und Poliklinik für Innere Medizin II, Klinikum rechts der Isar, TU München, München, Deutschland; 12 Klinik für Herzchirurgie, Universitätsklinikum Düsseldorf, Düsseldorf, Deutschland; 13 Klinik für Thorax- und Kardiovaskularchirurgie, Herz- und Diabeteszentrum Nordrhein-Westfalen, Universitätsklinik der Ruhr-Universität Bochum, Bad Oeynhausen, Deutschland; 14 Klinik für Kardiologie und Internistische Intensivmedizin, Asklepios Klinik St. Georg, Hamburg, Deutschland; 15 Klinik für Anästhesiologie, Intensiv‑, Notfallmedizin und Schmerztherapie, ECMO-Zentrum, Klinikum Kassel, Kassel, Deutschland; 16 Abteilung für Kardiologie und internistische Intensivmedizin, Asklepios Klinik Altona, Hamburg, Deutschland

**Keywords:** ECMO, COVID-19, Ressourcenknappheit, Priorisierung, Onlineumfrage, Indikationsstellung, Extracorporeal membrane oxygenation, COVID-19, Resource limitations, Prioritization, Online survey, Indication

## Abstract

**Hintergrund:**

Zu Beginn der Coronaviruspandemie wurde eine Überlastung der verfügbaren intensivmedizinischen Ressourcen befürchtet. Vielerorts wurden Routineeingriffe eingeschränkt und Kriterien für die Zuteilung knapper Ressourcen formuliert. In Deutschland kam es regional im Verlauf der Pandemie zeitweise zu Überlastungssituationen in den Kliniken. Speziell auf Intensivstationen zeigte sich eine Ressourcenknappheit, die zur Einschränkung von Leistungen und zu einer strengeren Indikationsstellung für ressourcenintensive Maßnahmen wie der extrakorporalen Membranoxygenierung (ECMO) geführt haben kann. Ziel dieser Arbeit ist es, einen Überblick über den Umgang mit diesen Belastungen an großen ECMO-Zentren in Deutschland zu gewinnen.

**Methodik:**

Über persönliche Ansprache wurde im Frühjahr 2021 je ein Vertreter an großen ECMO-Zentren in Deutschland zur Teilnahme an einer Onlineumfrage eingeladen.

**Ergebnisse:**

Insgesamt wurden 34 Einladungen verschickt, die Umfrage wurde von 23 Teilnehmern beantwortet. In allen Zentren wurden im Verlauf der Pandemie Routineeingriffe verschoben. Die Hälfte der Zentren erhöhte die Anzahl von Betten, auf denen ECMO-Verfahren durchgeführt werden konnten, in einem Drittel der Zentren wurde dennoch der Beginn mindestens einer ECMO-Unterstützung wegen einer befürchteten Ressourcenknappheit verzögert. In 17 % der Zentren wurde mindestens einem Patienten eine ECMO verweigert, die der Patient unter den Bedingungen vor der Pandemie aller Voraussicht nach erhalten hätte.

**Diskussion:**

Die Ergebnisse dieser Onlineumfrage zeigen, dass die erlebten Belastungen und Ressourcenengpässe in einigen Zentren zu einer zurückhaltenden ECMO-Indikationsstellung führten.

**Zusatzmaterial online:**

Zusätzliche Informationen sind in der Onlineversion dieses Artikels (10.1007/s00063-022-00951-3) enthalten.

## Abstract (en)

**Background:**

At the onset of the coronavirus pandemic, concerns were raised about sufficiency of available intensive care resources. In many places, routine interventions were postponed and criteria for the allocation of scarce resources were formulated. In Germany, some hospitals were at times seriously burdened during the course of the pandemic. Intensive care units in particular experienced a shortage of resources, which may have led to a restriction of services and a stricter indication setting for resource-intensive measures such as extracorporeal membrane oxygenation (ECMO). The aim of this work is to provide an overview of how these pressures were managed at large ECMO centers in Germany.

**Methods:**

One representative of each major ECMO referral center in Germany was invited to participate in an online survey in spring 2021.

**Results:**

Of 34 invitations that were sent out, the survey was answered by 23 participants. In all centers, routine procedures were postponed during the pandemic. Half of the centers increased the number of beds on which ECMO procedures could be offered. Nevertheless, in one-third of the centers, the start of at least one ECMO support was delayed because of a feared resource shortage. In 17% of centers, at least one patient was denied ECMO that he or she would have most likely received under prepandemic conditions.

**Conclusion:**

The results of this online survey indicate that the experienced pressures and resource constraints led some centers to be cautious about ECMO indications.

Zu Beginn des Jahres 2020 breitete sich SARS-Coronavirus Typ 2 („severe acute respiratory syndrome coronavirus type 2“, SARS-CoV-2), der Erreger der Coronaviruserkrankung 2019 („coronavirus disease 2019“, COVID-19) pandemisch aus. In vielen Ländern wurde befürchtet, dass der aufkommende Bedarf für medizinische Versorgung die verfügbaren Ressourcen übersteigen könnte. Im Hinblick auf die erwartete hohe Zahl besonders schwer erkrankter Patienten, die eine intensivmedizinische Versorgung brauchen würden, stieg auch in Deutschland die Sorge, dass Betten auf Intensivstationen, das notwendige intensivmedizinische Personal, spezifische Medikamente, aber auch Beatmungsgeräte und Geräte und Verbrauchsmaterialien zur extrakorporalen Membranoxygenierung (ECMO) nicht in ausreichendem Maß verfügbar sein könnten.

## Einführung

Die befürchtete Überlastung des Gesundheitssystems führte dazu, dass frühzeitig Kriterien und Leitlinien für die Zuteilung und Priorisierung knapper Ressourcen formuliert wurden [1–4]. Die stationäre und intensivmedizinische Versorgung einer zeitweise hohen Anzahl von Patienten mit COVID-19 hatte auch Auswirkungen auf die Versorgung von Patienten, die nicht an COVID-19 erkrankt waren. Viele Krankenhäuser schränkten ihre Kapazitäten zur Versorgung von Routineeingriffen ein. Aus Angst vor Infektionen mit SARS-CoV‑2 vermieden Patienten, akutmedizinische Behandlungen in Anspruch zu nehmen [5].

Die Behandlung von Patienten mit ECMO ist komplex und ressourcen- sowie personalintensiv und wird deshalb überwiegend an wenigen hierfür besonders spezialisierten Zentren durchgeführt [6]. Auch in diesen Zentren, häufig Krankenhäuser der Maximalversorgung, war die Belastung zu Beginn der Pandemie sehr hoch und der Bedarf notwendiger ECMO-Unterstützungen überstieg zeitweise an einigen Orten die im Regelbetrieb verfügbaren Ressourcen, sodass diese ausgeweitet werden mussten und Vorbereitungen für eine Priorisierung getroffen wurden [7, 8]. In Deutschland kam es landesweit betrachtet im Verlauf der COVID-19-Pandemie bisher nicht zu einer relevanten Überlastung des Gesundheitssystems. Im Fall lokaler oder regionaler Versorgungsengpässe und drohender Überlastungen konnte durch regionale und überregionale Verlegungen von Patienten weitgehend gewährleistet werden, dass die Versorgung auf dem etablierten Niveau aufrechterhalten werden konnte [9–12].

Wenngleich die vorliegenden Leitlinien zur Priorisierung verfügbarer Ressourcen nicht angewendet werden mussten, ist zu vermuten, dass bereits die Erwartung einer drohenden Ressourcenknappheit und Überlastung dazu geführt hat, dass Leistungen vorsorglich eingeschränkt wurden und Indikationsstellungen für ressourcenintensive Maßnahmen, wie maschinelle Beatmung und ECMO, sowohl für COVID-19-Patienten als auch für Patienten mit anderen Krankheitsbildern strenger und zurückhaltender erfolgten.

Vor diesem Hintergrund haben wir nach dem Ende der zweiten COVID-19-Welle im Frühjahr 2021 eine Onlineumfrage an großen ECMO-Zentren in Deutschland durchgeführt, um einen Überblick über den Umgang mit den vor- und zurückliegenden Belastungen zu bekommen.

## Methodik

Große ECMO-Zentren in Deutschland wurden zu einer Onlineumfrage eingeladen. Die Einladung für die Teilnahme an der Studie wurde jeweils an einen ärztlichen Vertreter pro Zentrum von den Autoren der aktuellen Arbeit persönlich per E‑Mail versandt und ein Link zur Umfrage zur Verfügung gestellt. Die Auswahl der ECMO-Zentren erfolgte über persönliche Kontakte der Autoren, die über ein breites Netzwerk zu ECMO-Zentren in Deutschland verfügen.

Für die Umfrage wurde ein Fragebogen über REDCap (REDCap Consortium, https://projectredcap.org/) erstellt. Um eine mögliche Ausweitung der Studie über den deutschsprachigen Raum hinaus zu ermöglichen, waren alle Fragen in englischer Sprache verfasst, eine deutsche Übersetzung wurde nicht angeboten. Die Beantwortung der Fragen erfolgte anonym über die REDCap-Datenbank. Es wurde nur ein minimaler Datensatz bezüglich des Zentrums abgefragt, eine Rückverfolgung der individuellen Antworten zu den jeweiligen Zentren ist nicht möglich. Der Bearbeitungszeitraum wurde auf 21 Tage nach Versand der ersten Einladung festgesetzt. Aufgrund der anonymen Datenerhebung ohne Speicherung von Daten, die eine Identifizierung der Probanden erlauben würden, war nach Rückfrage mit der Ethikkommission der Universität Freiburg eine ethische Beratung nicht notwendig (Antwort der Ethikkommission der Universität Freiburg per E‑Mail vom 08.03.2021 auf eine Anfrage vom selben Tag).

Eine Auswertung erfolgte nur auf Frageebene und nicht auf Zentrumsebene, um die Anonymität der einzelnen Teilnehmer zu sichern. Auf statistische Tests wurde bei geringer Fallzahl verzichtet. Die Ergebnisse sind daher rein deskriptiv und explorativ zu bewerten und erlauben keine Rückschlüsse über kausale Zusammenhänge.

Bei dieser Studie – auf Grundlage einer Onlineumfrage – wurden folgende Maßnahmen getroffen, um mögliche Verzerrungen (engl. „bias“) zu vermeiden, die die Aussagekraft der Beobachtungen einschränken könnten. Um eine möglichst hohe Antwortquote auf die versandten Einladungen zu erzielen, wurden die Adressaten durch persönliche Kontaktaufnahme zur Teilnahme an der Studie aufgefordert. Es wurde jeweils nur ein Vertreter pro Zentrum kontaktiert, um Doppelantworten zu verhindern. Der Umfang des Fragebogens wurde stark begrenzt und es wurden nur wenige dringend benötigte zentrumspezifische Daten erfragt, um eine Rückverfolgung unmöglich zu machen und die Bereitschaft zur wahrheitsgemäßen und vollständigen Dateneingabe zu erhöhen.

## Ergebnisse

Es wurden 34 Einladungen versendet und die Umfrage wurde von Vertretern aus 23 Zentren (68 %) beantwortet. Eine Liste der angefragten Zentren ist im Supplement einsehbar. Ebenso sind alle Fragen mit den jeweiligen Antworten im Supplement offengelegt.

### Zentrumscharakterisika

Von den befragten Zentren verfügten 22 von 23 (95,7 %) über eine Bettenkapazität von über 500 Betten. Im Jahr 2019 (dem letzten Jahr vor Beginn der globalen Ausbreitung von SARS-CoV-2) führten 14 von 23 (60,9 %) mindestens 31 venovenöse (vv‑)ECMO-Unterstützungen durch und 15 von 23 (65,2 %) behandelten mindestens 31 Patienten mit venoarterieller (va‑)ECMO. Prähospitale ECPR wurde 2019 in 9 von 23 Zentren (39,1 %) angeboten und 15 von 23 Zentren (65,2 %) boten ECMO-Implantationen außerhalb der eigenen Zentren und einen anschließenden Transport der Patienten in das ECMO-Zentrum an (Tab. 1).

**Table 1:** 

	Zentren^a^
*Bettenzahl*	
≥ 500	22 (95,7)
< 500	1 (4,3)
*Patienten mit vv-ECMO im Jahr 2019*	
1–30	9 (39,1)
≥ 31	14 (60,9)
*Patienten mit va-ECMO im Jahr 2019*	
1–30	8 (34,8)
≥ 31	15 (65,2)
*ECPRs im Jahr 2019*	
1–30	19 (82,6)
≥ 31	4 (17,4)
*Prähospitale ECPR wurde 2019 angeboten?*	
Ja	9 (39,1)
Nein	14 (60,9)
*ECMO-Implantation außerhalb des ECMO-Zentrums und Transport der Patienten in das ECMO-Zentrum wurde 2019 angeboten?*	
Ja	15 (65,2)
Nein	8 (34,8)

^a^Die Zahlen beschreiben die absolute Zahl an Zentren, für die der genannte Parameter zutrifft, sowie den relativen Anteil (%) in Bezug zur Gesamtkohorte (*n* = 23)

### ECMO-Zentren während der Coronaviruspandemie

Während der Pandemie wurden in allen befragten Zentren geplante Eingriffe und Operationen verschoben, davon in 10 von 23 Zentren (43,5 %) über einen Zeitraum von über 8 Wochen. Ebenso wurden in 10 von 23 Zentren (43,5 %) Notfallinterventionen verzögert, um die Patienten auf eine Infektion mit SARS-CoV‑2 zu testen. Nach Angaben der Befragten war die Versorgung von Patienten auf 11 von 23 Intensivstationen (47,8 %) während der Pandemie im Allgemeinen insgesamt schlechter als davor. In 21 von 23 Zentren (91,3 %) gab es Coronavirusinfektionen beim Personal, in 5 von 23 Zentren (23,8 %) wurde eine Ansteckung von Mitarbeitenden während der Arbeit vermutet. Unter den Befragten gaben 7 von 23 (30,4 %) an, zwischenzeitlich im Maximum mehr als 10 % Krankenstand im Personal verzeichnet zu haben. Von einer Ressourcenknappheit auf der Intensivstation berichteten 10 von 23 (43,5 %), wobei besonders das Personal und persönliche Schutzausrüstung betroffen waren (Tab. 2).

**Table 2:** 

	Zentren^a^
*Elektive operative Eingriffe wurden verschoben?*	
Ja	23 (100)
Nein	0 (0)
*Elektive operative Eingriffe wurden über einen Zeitraum von mehr als 8 Wochen verschoben?*	
Ja	10 (43,5)
Nein	13 (56,5)
*Notfalleingriffe (z.* *B. Herzkatheter bei akutem Myokardinfarkt) wurden verzögert, um Patienten zuvor auf SARS-CoV‑2 zu testen?*	
Ja	10 (43,5)
Nein	13 (56,5)
*Auf der Intensivstation wurden während des Beobachtungszeitraums mehr als 50 COVID-19-Patienten versorgt?*	
Ja	19 (82,6)
Nein	4 (17,4)
*Während der Pandemie war die Versorgung auf der Intensivstation qualitativ schlechter als vor der Pandemie?*	
Ja	11 (47,8)
Nein	12 (52,2)
*Haben sich Mitarbeiter mit SARS-CoV‑2 infiziert (während der Arbeit oder auch außerhalb)?*	
Ja	21 (91,3)
Nein	2 (8,7)
*Haben sich Mitarbeiter während der Arbeit mit SARS-CoV‑2 infiziert?*	
Ja	5 (23,8)
Nein	18 (76,2)
*Zu einem Zeitpunkt während der Pandemie waren mehr als 10* *% der Mitarbeiter erkrankt?*	
Ja	7 (30,4)
Nein	16 (69,6)
*Kam es zu einem Zeitpunkt während der Pandemie zu einem kritischen Mangel an wichtigen benötigten Ressourcen, der einen negativen Einfluss auf die Versorgungsqualität hatte?*	
Ja	10 (43,5)
– Knappheit spezifischer Medikamente	8 (34,8)
– Personalmangel	9 (39,1)
– ECMO-Equipment	7 (30,4)
– Persönliche Schutzausrüstung	9 (39,1)
Nein	13 (56,5)

^a^Die Zahlen beschreiben die absolute Zahl an Zentren, für die der genannte Parameter zutrifft, sowie den relativen Anteil (%) in Bezug zur Gesamtkohorte (*n* = 23)

### ECMO-Unterstützung während der Coronaviruspandemie

In 8 von 22 Zentren (36,4 %) wurden die Kriterien zur Indikation einer vv-ECMO während der Pandemie geändert. 5 von 23 (21,7 %) setzten ein etabliertes Angebot für ECMO-Implantationen außerhalb des eigenen Zentrums aus, jedoch führten ebenso viele Zentren, die einen solchen Service vor Beginn der COVID-19 Pandemie nicht angeboten hatten, diesen während der Pandemie ein. Insgesamt erhöhten 13 von 23 Zentren (56,5 %) die Anzahl von Betten, auf denen ECMO-Verfahren durchgeführt werden konnten, und 13 von 23 Zentren (56,5 %) setzten Mitarbeiter zur Versorgung von Patienten mit ECMO ein, die zuvor nicht für die Versorgung von ECMO-Patienten zuständig waren.

In 7 von 23 Zentren (30,4 %) wurde der Beginn mindestens einer ECMO-Unterstützung wegen einer befürchteten Ressourcenknappheit verzögert und in 4 von 23 Zentren (17,4 %) wurde mindestens einem Patienten eine ECMO verweigert, die dieser Patient unter den Bedingungen vor der Pandemie aller Voraussicht nach erhalten hätte. Ebenso hat in 4 von 23 Kliniken (17,4 %) mindestens einmal ein Patient eine extrakorporale Reanimation (ECPR) nicht erhalten unter Bedingungen, unter denen der Patient vor der Pandemie voraussichtlich eine ECPR erhalten hätte. In keinem der befragten Zentren wurde eine ECMO explantiert, um die Konsole für einen anderen Patienten bereitzustellen. 8 von 23 (34,8 %) Zentren erarbeiteten eine zentrumsspezifische Ethikleitlinie für die ECMO-Therapie (Tab. 3).

**Table 3:** 

	Zentren^a^
*Indikationskriterien für vv-ECMO wurden verändert*^b^	
Ja	8 (36,4)
Nein	14 (63,6)
*Indikationskriterien für va-ECMO wurden verändert*	
Ja	1 (4,3)
Nein	22 (95,7)
*Indikationskriterien für ECPR wurden verändert*	
Ja	5 (21,7)
Nein	18 (78,3)
*ECMO-Implantation außerhalb des ECMO-Zentrums und Transport der Patienten in das ECMO-Zentrum wurde beendet?*	
Ja	5 (21,7)
Nein	18 (78,3)
*ECMO-Implantation außerhalb des ECMO-Zentrums und Transport der Patienten in das ECMO-Zentrum wurde begonnen oder ausgeweitet?*	
Ja	5 (21,7)
Nein	18 (78,3)
*Anzahl an ECMO-Betten wurden während der Pandemie erweitert?*	
Ja	13 (56,5)
Nein	10 (43,5)
*Eine Ressourcenknappheit für ECMO-Versorgung wurde befürchtet?*	
Ja	14 (60,9)
– Im Hinblick auf Personal	13 (56,5)
– Im Hinblick auf ECMO-Equipment	12 (52,5)
Nein	9 (39,1)
*ECMO-Unterstützungen wurden verzögert oder abgelehnt wegen einer befürchteten Ressourcenknappheit?*	
Ja	7 (30,4)
Nein	16 (69,6)
*Mindestens einmal wurde ECMO-Unterstützung aufgrund von Ressourcenknappheit rationiert?*	
Ja	4 (17,4)
Nein	19 (82,6)
*Mitarbeiter, die normalerweise nicht in der Versorgung von ECMO-Patienten tätig waren, wurden in der Versorgung von ECMO-Patienten eingesetzt?*	
Ja	13 (56,5)
Nein	10 (43,5)
*Mindestens einmal wurde einem Patienten eine ECMO unter Bedingungen verweigert, unter denen er vor der Pandemie eine ECMO erhalten hätte?*	
Ja	4 (17,4)
Nein	19 (82,6)
*Mindestens einmal wurde einem Patienten eine ECPR unter Bedingungen verweigert, unter denen er vor der Pandemie eine ECMO erhalten hätte?*	
Ja	4 (17,4)
Nein	19 (82,6)
*Mindestens einmal wurde bei einem Patienten eine ECMO beendet, um einem anderen Patienten eine ECMO bereitstellen zu können?*	
Ja	0 (0)
Nein	23 (100)
*Am Zentrum gab es eigene Ethikrichtlinien für die ECMO-Unterstützung während der Pandemie*	
Ja	8 (34,8)
Nein	15 (65,2)

^a^Die Zahlen beschreiben die absolute Zahl an Zentren, für die der genannte Parameter zutrifft, sowie den relativen Anteil (%) in Bezug zur Gesamtkohorte (*n* = 23)

^b^*n* = 22 (wegen einer fehlenden Antwort)

## Diskussion

Diese Umfrage, die von ärztlichen Mitarbeitern an 23 großen ECMO-Zentren in Deutschland beantwortet wurde, gibt einen umfassenden Überblick über erwartete und eingetretene Versorgungsengpässe im ersten Jahr der COVID-19-Pandemie in Deutschland und über den Einfluss auf das Angebot von vv- und va-ECMO und ECPR.

In 14 Zentren, 60,9 % aller teilnehmenden Zentren, wurden im letzten Jahr vor der Pandemie jeweils mehr als 30 vv- und va-ECMO implantiert. Laut einer aktuellen Erhebung implantieren nur 27 Zentren Deutschland mehr als 50 ECMO (vv plus va) pro Jahr und nur 53 Zentren mehr als 21 [6]. Wir können daher davon ausgehen, dass viele der großen ECMO-Zentren an dieser Umfrage teilgenommen haben.

Vor dem Hintergrund von Berichten über zeitweise schwerwiegende regionale Überlastungen leistungsfähiger Gesundheitssysteme, wie z. B. in Norditalien oder in New York, entwickelte sich auch in Deutschland im Frühjahr 2020 die Sorge, dass es zu Überlastungen des Gesundheitssystems kommen werde [13, 14]. In vielen Kliniken wurden Notfallpläne entwickelt oder aktiviert und die Kapazität zur intensivmedizinischen Versorgung von Patienten wurde erweitert [15]. Um eine ausreichende Zahl an Intensivbetten für Patienten bereitzustellen, die eine maschinelle Beatmung oder sogar ECMO benötigen würden, wurden Eingriffe und Operationen aufgeschoben [16]. Von allen an dieser Studie teilnehmenden ECMO-Zentren wurde ein solches Vorgehen bestätigt, jedoch wurden nur in 10 Zentren (43,5 %) Eingriffe mehr als 8 Wochen aufgeschoben. Neben einer Verzögerung von Eingriffen wurden aber auch Qualitätseinbußen in der medizinischen Versorgung während der Pandemie befürchtet und beobachtet. So wurden Notfalleingriffe verzögert, z. B. um Patienten vor diesen Eingriffen zunächst auf eine Infektion mit dem Coronavirus zu testen.

Bereits innerhalb der ersten Monate der Pandemie im Jahr 2020 wurden laut einer Schätzung weltweit mehrere Millionen Operationen verschoben [17]. Unabhängig davon nahm insbesondere zu Beginn der Pandemie die Häufigkeit von Notfallbehandlungen, die nicht im Zusammenhang mit COVID-19 standen (z. B. Herzinfarkte), zeitweise deutlich ab [18–20]. Dies ist am ehesten damit zu erklären, dass Patienten aus Angst, sich im Krankenhaus mit SARS-CoV‑2 zu infizieren, sich auch mit Beschwerden, mit denen sie sich vormals zur Notfallbehandlung in einem Krankenhaus vorgestellt hätten, nicht in ärztliche Behandlung begaben [21].

Zu Beginn der Pandemie waren Unsicherheiten und Ängste vor einer Ansteckung mit dem Virus weit verbreitet, auch beim medizinischen Personal [22]. Vor der Verfügbarkeit wirksamer Impfstoffe und oftmals unter einem Mangel an medizinischer Schutzausrüstung infizierten sich zahlreiche Ärzte und Pflegekräfte mit SARS-CoV‑2 und einige verstarben an COVID-19 [23, 24]. In dieser Umfrage berichtete etwa ein Viertel der Befragten von Mitarbeitern, die sich während der Arbeit mit dem Coronavirus infiziert hatten. Jedoch nur in etwa einem Drittel der Kliniken fielen zu einem Zeitpunkt während der Pandemie mehr als 10 % der Mitarbeitenden aufgrund von Krankheit aus. Serologische Daten einer Untersuchung für denselben Zeitraum wie für diese Umfrage legten eine Seroprävalenz von Antikörpern gegen SARS-CoV‑2 von 5,1 % unter Krankenhausmitarbeitern nahe [25]. Trotz des geringen berichteten Mitarbeiterausfalls beschrieben etwa 40 % der befragten Zentren, dass ein kritischer Personalmangel zeitweise einen negativen Einfluss auf die Versorgungsqualität hatte. Da zudem in über 50 % der Zentren nichtfachspezifische Mitarbeiter zur Versorgung von ECMO-Patienten eingesetzt wurden, ist von einer Qualitätseinbuße im Rahmen der ECMO-Therapie auszugehen.

Vor dem Hintergrund eines erwarteten Ressourcenmangels wurden zu Beginn der Pandemie Kriterien und Abläufe für die Allokation knapp werdender Ressourcen diskutiert [3, 8, 26]. Die Mehrzahl der Zentren, die an dieser Umfrage teilnahmen, behandelte im Jahr 2019, dem letzten Jahr vor Beginn der Pandemie, jeweils mehr als 30 Patienten mit vv- und mit va-EMCO-Unterstützung und hatten damit eine besonders große Erfahrung in der Durchführung dieser Verfahren [27, 28]. Im Hinblick auf die Versorgung mit ECMO wurden sowohl eine Erweiterung der Versorgungskapazitäten als auch eine Einschränkung des Angebots diskutiert, jeweils in Anhängigkeit von den Möglichkeiten und der Erfahrung der Zentren [7].

In dieser Umfrage beschrieb jeweils etwa die Hälfte der Zentren eine Erweiterung oder eine Reduktion ihrer Kapazitäten zur Behandlung mit ECMO. Während ein Fünftel der Zentren ein bestehendes System zur externen ECMO-Implantation mit anschließendem Transport der Patienten in das ECMO-Zentrum beendeten, etablierte eine ebenso große Zahl an Kliniken ein solches System während der Pandemie neu. Die Indikationen für vv- oder va-ECMO oder ECPR wurden nur in wenigen Zentren verändert, an keinem Zentrum wurde eine laufende ECMO-Unterstützung bei einem Patienten beendet, nur um einem anderen Patienten eine ECMO zur Verfügung stellen zu können. Dennoch beschrieb ein Drittel der Zentren Situationen, in denen eine ECMO aufgrund einer befürchteten Ressourcenknappheit abgelehnt oder aufgeschoben wurde. Bei 17 % der Zentren sei mindestens einmal einem Patienten eine ECMO oder eine ECPR verweigert worden unter Bedingungen, unter denen vor der Pandemie eine ECMO oder ECPR nicht abgelehnt worden wäre. Diese Beobachtungen lassen sich so zusammenfassen, dass allein die Erwartung möglicher Versorgungsengpässe (und nicht erst die Überlastung oder der manifeste Mangel) bereits zu einer strengeren und zurückhaltenden Indikationsstellung von ECMO geführt haben könnte.

Einige Limitationen sind bei der Interpretation der Ergebnisse dieser Studie zu beachten. Die Umfrage konzentrierte sich auf wenige ausgewählte Zentren mit großer Expertise in der Behandlung von Patienten mit ECMO. In Deutschland wird ECMO-Unterstützung jedoch an mehr als 500 Kliniken durchgeführt, daher lassen sich die Ergebnisse dieser Umfrage nicht für alle Behandlungen von ECMO-Patienten verallgemeinern [6]. Weiterhin bezieht sich die Umfrage auf Beobachtungen während der ersten beiden Wellen von COVID-Patienten in Deutschland von 2020 bis zum Frühjahr 2021. Zu Beginn der Pandemie lagen viele Informationen und Erfahrungen, die im weiteren Verlauf der Pandemie gewonnen werden konnten, noch nicht vor. Ebenso waren wirksame Impfstoffe gegen SARS-CoV‑2 in Deutschland erst ab Ende Dezember 2020 verfügbar, also erst am Ende des hier berichteten Zeitraums. Zuletzt ist zu beachten, dass die Umfrage anonymisiert durchgeführt wurde und daher die Antworten nicht im Kontext konkreter Situationen in einzelnen Kliniken bewertet werden können. Zukünftige Analysen sollten daher auch untersuchen, inwiefern die subjektiven Wahrnehmungen der Betroffenen mit objektiveren Faktoren übereinstimmen.

## Fazit

Die Ergebnisse der hier vorgestellten Onlineumfrage unter ärztlichen Vertretern großer ECMO-Zentren in Deutschland geben Hinweise darauf, dass bereits eine befürchtete Überlastung der verfügbaren Ressourcen (und nicht erst ein manifester Mangel) zu einer zurückhaltenden ECMO-Indikationsstellung führte. Diese Beobachtungen sollten bei der Aufstellung von Notfall- und Krisenplänen sowie im Management möglicher Krisensituationen berücksichtigt werden.

## Supplementary Information





