# Supplementary material for: ECMO support during the first two waves of the corona pandemic—a survey of high case volume centers in Germany
Source: Med Klin Intensivmed Notfmed. 2022 Sep 8;118(6):492–8. [Article in German] doi: 10.1007/s00063-022-00951-3 (PMC9453733; doi:10.1007/s00063-022-00951-3)
Supplement: Supplementary file 1 [file 63_2022_951_MOESM1_ESM.docx]

Asklepios Klinik Harburg, Kardiologie

Asklepios Klinik Nord, Kardiologie

Asklepios Klinik St. Georg, Kardiologie

Bad Oeynhausen, Klinik für Thorax- und Kardiovaskularchirurgie

Helios Klinikum Wiesbaden, Kardiologie

Katharinenhospital Stuttgart, Intensivmedizin

Klinikum Heilbronn, Kardiologie

Klinikum Ibbenbüren, Anästhesie

Klinikum Ludwigsburg, Intensivedizin

Ludwig-Maximilians-Universität München, Interdisziplinäre Internistische Intensivstation

Marienhospital Stuttgart, Anästhesie

Marienkrankenhaus Hamburg

Medizinische Hochschule Hannover, Herz-, Thorax-, Transplantations- und Gefäßchirurgie

Technische Universität München, internistischen Intensivstation

Uniklinik Aachen, Pneumologie

Uniklinik Düsseldorf, Herzchirurgie

Uniklinik Essen, Thorax- und Kardiovaskuläre Chirurgie

Uniklinik Freiburg, Kardiologie

Uniklinik Halle-Wittenberg, Kardiologie

Uniklinik Hamburg, Kardiologie

Uniklinik Hannover, Kardiologie

Uniklinik Heidelberg, Kardiologie

Uniklinik Köln, Herzchirurgie

Uniklinik Leipzig, Kardiologie

Uniklinik Magdeburg, Herzchirurgie

Uniklinik Mainz, Kardiologie

Uniklinik Regensburg, Anästhesie

Uniklinik Tübingen, Anästhesie

Uniklinik Ulm, Anästhesie/Herzchirurgie

Uniklinik Würzburg, Anästhesie
