# Supplementary material for: ECMO support during the first two waves of the corona pandemic—a survey of high case volume centers in Germany
Source: Med Klin Intensivmed Notfmed. 2022 Sep 8;118(6):492–8. [Article in German] doi: 10.1007/s00063-022-00951-3 (PMC9453733; doi:10.1007/s00063-022-00951-3)
Supplement: Supplementary file 2 [file 63_2022_951_MOESM2_ESM.pdf]

General questions

Dear ECMO friends fighting the coronavirus pandemic !

We would like to invite you to our survey on ECMO and ECPR during the SARS-CoV-2 pandemic.

The survey will takes 5-10 minutes to complete and features no question regarding patient data. Since the survey is anonymous, we would like to ask you to fill it only once. Alos, it should only be answered from one person per hospital, both would lead to bias.

All of the following questions regard your whole hospital if not stated otherwise.
The year 2019 is used as reference for the state bevore the coronaviurs pandemic. When more accurate data is available to you for 2018 or 2017, this data can be used insted.

1) Where is your hospital geographically located?
- ◯ Europe
- ◯ Asia
- ◯ America (North or South)
- ◯ Africa

2) How many beds are there in your hospital (total number of beds)?
- ◯ < 500 beds
- ◯ 500-1000 beds
- ◯ >1000 beds

3) How many V-V ECMO treatments have been performed at your hospital in 2019 (number of patients treated with V-V ECMO)?
- ◯ < 10
- ◯ 10-30
- ◯ 31-50
- ◯ >50

4) How many V-A ECMO treatments (except ECPR) have been performed at your hospital in 2019?
- ◯ < 10
- ◯ 10-30
- ◯ 31-50
- ◯ >50

5) How many ECPRs have been performed at your hospital in 2019?
- ◯ < 10
- ◯ 10-30
- ◯ 31-50
- ◯ >50

6) Did you hospital offer prehospital ECPR service in 2019?
- ◯ Yes
- ◯ No

7) Did your hospital offer ECMO retrieval (V-A or V-V) in 2019?
- ◯ Yes
- ◯ No

Corona pandemic questions

Please complete the survey below.

Thank you!

Questions regarding your hospital during the coronavirus pandemic.

Were elective surgeries or interventions deferred or cancelled at your hospital for organizational reasons due to the corona pandemic?	◯ Yes<br>◯ No
if so, during which time period?	◯ < 1 week<br>◯ 1-4 weeks<br>◯ 4-8 weeks<br>◯ >8 weeks
During the corona pandemic, were urgent treatments (e.g., operations or emergency interventions, such as coronary angiography in myocardial infarction) ever delayed in order to test a patient for SARS-CoV-2 prior to the intervention/surgery?	◯ Yes<br>◯ No

Questions regarding your ICU during the coronavirus pandemic.

How many COVID-19 patients have been treated on your ICU in total, so far?	◯ < 10<br>◯ 10-30<br>◯ 31-50<br>◯ >50
The general quality of ICU treatment during the corona pandemic for non-covid patients when compared to the treatment quality in 2019 was ... compared to 2019.	◯ worse<br>◯ little worse<br>◯ unchanged<br>◯ little better<br>◯ much better
Was personnel working on your ICU (physicians, nurses or others) infected with SARS-CoV2? Both, infections contract at work or outside the hospital are counted.	◯ Yes<br>◯ No
Please estimate the ratio of sick leave at the highest point during the pandemic (percentage; including physicians, nurses or others).	◯ 0%<br>◯ 1-5%<br>◯ 6-10%<br>◯ 11-20%<br>◯ 21-50%<br>◯ >50%
Do you believe that a significant amount of infections with SARS-CoV-2 in your staff occured during work?	◯ Yes<br>◯ No
Did you experience critical resource or equipment shortages with an influence on the quality of care for patients or on the safety of personnel during the coronavirus pandemic on your ICU?	◯ Yes<br>◯ No
Did the shortage include the supply with medical consumables or drugs?	◯ Yes<br>◯ No

Did the shortage include the workforce (nurses, physicians or other personnel)?	◯ Yes<br>◯ No
Did the shortage include ECMO equipment?	◯ Yes<br>◯ No
Did the shortage include personnel safety equipment (e.g. FFP 2/3 mascs etc.)?	◯ Yes<br>◯ No

ECMO spesific question

Please complete the survey below.

Thank you!

Questions regarding ECMO indications.

Did you change criteria for V-V ECMO in non-COVID-19 patients during the coronavirus pandemic?
- ◯ Yes
- ◯ No

Did you change criteria for V-A ECMO (excluding ECPR) during the coronavirus pandemic?
- ◯ Yes
- ◯ No

Did you change criteria for ECPR during the coronavirus pandemic?
- ◯ Yes
- ◯ No

Did you stop any of the established ECMO services (e.g., prehospital ECPR or ECMO retrieval) at your hospital during the coronavirus pandemic?
- ◯ Yes
- ◯ No

Did you expand or start ECMO services (e.g., ECMO retrieval, counting V-V or V-A or ECPR) during the pandemic not offered at your hospital in 2019?
- ◯ Yes
- ◯ No

Did you change the number of beds or treatment units available for ECMO in reaction to the coronavirus pandemic?
- ◯ Yes
- ◯ No

Were the number of ECMO capable beds or units increased or reduced?
- ◯ increased by >50%
- ◯ increased by 1-50%
- ◯ reduced by 1-50%
- ◯ reduced by >50%

ECMO organization and resources facing the coronavirus pandemic.

Did you fear or expect relevant resource limitations with an effect on ECMO treatment on your ICU?
- ◯ Yes
- ◯ No

Were you worried about workforce limitations?
- ◯ Yes
- ◯ No

Were you worried about a shortage in ECMO equipment (consoles, ECMO tubing, etc.)?
- ◯ Yes
- ◯ No

Did you delay or withhold ECMO (V-V or V-A) therapy due to an expected lack of resources during the pandemic in at least one patient?
- ◯ Yes
- ◯ No

If so, which type of ECMO did it affect?
- ◯ V-V ECMO only
- ◯ V-A ECMO only
- ◯ both

Have you been in a situation on your ICU in which you had to ration/terminate ECMO (V-V or V-A) due to actual shortage in at least one situation?
- ◯ Yes
- ◯ No

if so, which type of ECMO did it affect?	◯ V-V ECMO only<br>◯ V-A ECMO only<br>◯ both

Impact on individual patients.

Did you allocate medical personnel not regularly treating ECMO patients for the treatment of ECMO patients during the pandemic?	◯ Yes<br>◯ No
Was ECMO (V-V or V-A, excluding ECPR) withheld from any patient during the pandemic that would have received it according to your standards of 2019?	◯ Yes<br>◯ No
Was ECPR withheld from any patient during the pandemic that would have received ECPR according to your standards of 2019?	◯ Yes<br>◯ No
Was ECMO (V-V or V-A) withdrawn from a patient during the pandemic to give the ECMO to another patient at least once?	◯ Yes<br>◯ No
Were center specific ethical guidelines or algorithms concerning ECMO treatment (including triage) established during the corona pandemic at your institution?	◯ Yes<br>◯ No
